# How different advertising appeals (green vs. non-green) impact consumers' willingness to pay a premium for green agricultural products

**DOI:** 10.3389/fpsyg.2022.991525

**Published:** 2022-09-20

**Authors:** Manhua Zheng, Decong Tang, Jianhong Chen, Qiujin Zheng, Anxin Xu

**Affiliations:** ^1^Department College of Economics and Management, Organization Fujian Agriculture and Forestry University, Fuzhou, China; ^2^Department School of Journalism and Communication, Organization Minjiang University, Fuzhou, China

**Keywords:** advertising appeals, green agricultural products, willingness to pay a premium, green perceived value, self-construal

## Abstract

Green food has exceptional impacts in addressing food safety and environmental challenges. However, consumers' perception of green food is not substantial, which results in a decline in consumption intention. Since advertising appeals can play a bridging role in resolving information asymmetry. This study is based on self-construal theory, chooses green agricultural products images and text as experimental stimuli, and analyzes the interaction and influence mechanism between advertising appeals and consumers' willingness to pay a premium for green agricultural products through three sets of experimental studies. The findings demonstrate that self-construal and green agricultural product advertising appeals interact to influence consumers' willingness to pay a premium for green agricultural products. Green perceived value is more strongly influenced by matching dependent self-construal and green advertising appeals than non-green advertising appeals. Green perceived value plays a full mediating role in this interactive effect. Green agricultural products companies should adopt different advertising strategies according to the various categories of consumers to enhance consumers' green perceived value and increase the willingness to pay a premium.

## 1. Introduction

Environmental problems (such as global warming, water and soil pollution, energy shortages, etc.) and food safety concerns have become more prevalent. The probability of extreme heat events, such as the one in South Asia this year, was approximately once every 3,000 years before human-caused climate change began, according to research published on May 23 by Frederick Otto of the Grantham Research Institute for Climate Change and the Environment at Imperial College London, UK (HuanQiu, 2022). High temperatures have worsened air pollution, with “bad” air quality ratings on June 17 in several French cities like Paris, Lille, and Marseille (NetEase, 2022). Green consumerism can aid in lowering carbon emissions, which are blamed for “excessive carbon emissions” that cause “global warming.”

Green food is quite effective at lowering pollution levels. In China, in comparison to traditional agricultural production methods, the green food production model has reduced chemical nitrogen fertilizer application by 39%, reduced pesticide use intensity by 60%, and increased soil organic matter content by 17.6%, according to the report “Evaluation of Ecological and Environmental Effects, Economic Benefits, and Social Effects of Green Food.” From 2009 to 2018, there was an overall decrease of 14.58 million tons in chemical nitrogen fertilizer input, 542,000 tons in pesticide input, and 55.58 million tons in carbon dioxide emissions. Then resulted in an overall value of 3.2 trillion yuan in ecosystem services (CGFDC, 2020).

Environmentally friendly products, such as those that are green and organic, are referred to as “green agricultural products” (Zhou et al., 2017). Green agricultural products are becoming more and more popular among customers as their income, environmental consciousness, and worry about food safety and environmental pollution incidents rise (Han et al., 2010; Wang and Wang, 2010; Chen et al., 2015). However, consumers' lack of knowledge of green agricultural products and mistrust of corporate advertising limits their desire to consume (Rana et al., 2017; Jiang et al., 2021). Companies are increasingly focused on how to enhance their reputation as environmentally friendly businesses in the eyes of consumers (Luo et al., 2020; Islam and Hussain, 2022). The key to solving these problems lies in how to increase consumers' attention to green agricultural products, enhancing consumers' willingness to pay green, and encouraging consumers to pay for premium green agricultural products.

There are three primary categories of the current study on willingness to pay a premium for green agricultural products: individual characteristics aspects (gender, education, income, age, marital status, etc.; Zhang and Wang, 2009; de Medeiros et al., 2016; Kucher et al., 2019; Yang et al., 2021), internal psychological factors (trust, cognition, perception, environmental beliefs, health awareness, signaling, peer effects, etc.; de Medeiros et al., 2016; Salazar and Oerlemans, 2016; Konuk, 2018; Zhang et al., 2018; Berger, 2019; Zhong and Chen, 2019; Xu and Lin, 2021), external contextual aspects (market environment, environmental and social attributes, policies, etc.; Min et al., 2017; Kucher et al., 2019; Shao and Unal, 2019) can affect the amount of premium paid by consumers.

The aforementioned studies could yet use some further development. Firstly, the introduction of advertising appeals can compensate for the existing studies on the information asymmetry in the market for produce, the lack of consumer knowledge and trust in green agricultural products, and the fact that current studies rarely use it as the research object. Second, it's unclear if advertising appeals—green vs. non-green—can raise consumers' willingness to pay a premium. Third, more research on boundary circumstances is still required. Advertising appeals that are matched to customer attributes have been found to affect consumers' purchasing decisions and actions (Wang et al., 2021). Self-construal is regarded as being connected to a consumer's willingness to pay as one of the key consumer traits (Dogan and Ozmen, 2019). Through verbal or sensory cues, advertisements shape consumers' attitudes and actions toward certain products or services (Pileliene and Grigaliunaite, 2017). Advertising appeals are a significant external element influencing consumer behavior, according to Yang et al. (2015) and Sheng et al. (2019). Then, under what circumstances may advertising appeals maximize the impact of advertising and inspire customers to pay a premium?

Based on the analysis above, advertising appeals help to enhance consumers' attention to the quality of green agricultural products, reduce consumers' search costs and switching costs (Pozzi, 2012; Richards et al., 2017), enhance consumers' knowledge of the standards of green agricultural products production, processing, transportation, packaging, and the costs paid by companies, which in turn affects consumers' perceived green value and ultimately enhances consumers' willingness to pay a premium (Jiang et al., 2021). Therefore, the study uses green agricultural products as its research object and conducts three sets of experiments to examine the mechanisms influencing consumers' willingness to pay a premium for green agricultural products and the mediating role played by green perceived value, to provide ideas for governments and enterprizes to promote green consumption and alleviate environmental problems.

## 2. Literature review and hypotheses

### 2.1. Advertising appeal and willingness to pay a premium for green agricultural products

Advertising appeal is the process through which a business informs consumers about a good or service using advertising techniques to draw them in and encourage consumer spending (Akbari, 2015). The technique through which businesses encourage customers to make purchases by using advertising content that promotes green messages is known as “green advertising appeal” (Carlson et al., 1996). Based on the compilation of previous studies, green advertising appeals can be classified into the following four categories.

First, green advertising appeals can be divided into egoistic advertising appeal and altruistic advertising appeal based on the target audience of the advertisement (Song and Kim, 2019). A relevant study based on the construal level theory (CLT) showed that when promoting organic foods, matching illustrations (with photos) with altruistic appeal would improve advertising effectiveness. When promoting conventional foods, matching photos (with illustrations) with altruistic appeal would improve advertising effectiveness (Septianto et al., 2019). Related research based on information processing fluency theory suggested that under egoistic advertising appeal, cool anthropomorphic images could lead to higher purchase intention for green products due to the mediating role of trust in brand competence. However, under altruistic advertising appeal, cute anthropomorphic images could enhance consumers' trust in brand goodwill and make them more willing to purchase green products (Lu et al., 2021).

Second, according to the level of detail of advertising content, green advertising appeals can be divided into concrete appeal and abstract appeal (Yang et al., 2015). According to CLT, when a green product was related to the interests of others, consumers would think that the product was psychologically distant from themselves, and consumers tended to think using abstraction, and the abstract appeal was more likely to generate green purchase intentions; while when a green product was related to the interests of the self, neither abstract appeal nor concrete appeal could have an impact on green purchase intentions. Among them, public self-awareness and identity salience moderated the effects of advertising appeals and interest associations on green purchase intention (Yang et al., 2015).

Third, based on consumer characteristics, green advertising appeals can be divided into emotional advertising appeal and rational advertising appeal (Matthes et al., 2014). Research on high school students who smoked showed that for emotional advertising appeal, narrative self-reference led to anti-smoking behavioral intentions more than analytical self-reference; for rational advertising appeal, analytical self-reference led to anti-smoking behavioral intentions more than narrative self-reference (Lee et al., 2020). A related study on smartphones showed that emotional advertising appeal had a positive effect on hedonism and rational advertising appeal had a positive effect on utilitarian values (Kim et al., 2020).

Fourth, according to the types of advertisements, green advertising appeals can be divided into green advertising appeal and non-green advertising appeal (Ku et al., 2012), with green advertising appeal emphasizing the attributes of products or services related to environmental friendliness and non-green advertising appeal emphasizing the attributes of products or services related to consumer benefits, including health and saving money (Iyer and Banerjee, 1993). Only a few pertinent research have been conducted on the fourth category, focusing mostly on how advertising appeals affect purchase intention, advertising attitude, product attitude, and persuasiveness (Schuhwerk and Lefkoff-Hagius, 1995; Ku et al., 2012; Kong and Zhang, 2014; Yang et al., 2017; Sun and Miao, 2018; Yuan et al., 2020). Research on how advertising appeals affect consumers' willingness to pay a premium for green agricultural products is lacking. Therefore, this study is based on the signaling theory and aims to investigate the impact of advertising appeals on willingness to pay a premium for green agriculture products.

Green agricultural products have a good reputation, which can improve consumer experiences. However, consumers find it challenging to understand their quality and production processes, which causes them to question the quality of green agricultural products. As a result, there may be communication and transactional issues between businesses and consumers (Ki and Kim, 2022). “Trusted signals” (green or organic food certification) can lessen this information asymmetry because it is more expensive for certified products to violate the applicable standards and laws, forcing businesses to focus on product quality to survive in the market. This separation of high- and low-quality producers makes “trusted signals” more effective (Atkinson and Rosenthal, 2014). In an asymmetric information environment, customers eventually suffer the expense of obtaining the signal and pay a premium for green agricultural products. Consumers rely on signals to identify products to generate internal impressions of green agricultural products (Yang et al., 2021).

Due to their own potential desire to purchase, consumers may be willing to pay a premium for green agricultural products. This desire to purchase can be sparked by external environmental stimuli, while the company's advertising, the delivery of product information, the color of the product's packaging, and the transparency of the packaging can influence consumers' green consumption behavior, and these external stimuli are all relevant to advertising appeals (Owusu and Anifori, 2013; Septianto et al., 2019; Srivastava et al., 2019; Biondi and Camanzi, 2020). Willingness to pay a premium for green agricultural products due to green advertising appeals indicates their willingness to do so for reasons of environmental (social) interest considerations. Consumers' willingness to pay a premium for green agricultural products to meet their dietary demands are referred to as “willingness to pay a premium for green agricultural products due to non-green advertising appeal.” Consequently, the following study hypotheses are put out.

**H1**. Advertising appeals significantly increase consumers' willingness to pay a premium more for green agricultural products. Green advertising appeal has a more considerable impact on consumers' willingness to pay a premium for green agricultural products than non-green advertising appeal.

**H1a**. Green advertising appeal significantly increase consumers' willingness to pay a premium for green agricultural products.

**H1b**. Non-green advertising appeal significantly increase consumers' willingness to pay a premium for green agricultural products.

### 2.2. Advertising appeal and green perceived value

Consumers' perceptions of green agricultural products are distinct and vary depending on the advertising appeals they are exposed to Xue et al. (2019). Scarcity advertising appeal (non-green) helped to enhance consumers' perceived value (Eisend, 2008). Emotional green appeal (the proud green appeal and the admiring green appeal) contributed to increasing consumers' perceived value (Wang et al., 2017). Specifically, positive green emotional appeal made consumers feel happy, helped increase consumer acceptance of the advertisement and brand (Fredrickson, 2001), and thus increased consumers' green perceived value (Wang et al., 2022). Consumers' green perceived value less strongly because non-green advertising appeal focuses more on characteristics connected to how green agricultural products perform (Kong and Zhang, 2014). Consumers can more clearly detect a higher level of green perceived value thanks to green advertising appeal, which communicates more characteristics related to the environmental friendliness of green agricultural products (Kong and Zhang, 2014). Companies help consumers gain a deeper understanding of the environmentally friendly attributes of products and their contribution to sustainable development by informing consumers about these topics through advertisements (Zhang et al., 2010; Sheng et al., 2021), making it much easier for consumers to understand the green perceived value of goods and services. Consequently, the following study hypothesis is put out.

**H2**. Advertising appeals significantly increase the green perceived value of green agricultural products. Green advertising appeal has a larger impact on the green perceived value of green agricultural products than non-green advertising appeal.

### 2.3. Green perceived value and willingness to pay a premium for green agricultural products

According to consumer value theory, customers should consider more than just the characteristics of the items themselves. They should also consider the value perceptions that the products give consumers (Hur et al., 2013). The part of the green perceived value of green agricultural products related to the environmentally friendly characteristics and attributes that are acknowledged by consumers and are the reason why consumers are willing to pay a premium for green agricultural products is referred to as the green perceived value of green agricultural products (Yang and Zhou, 2006). Customers' demands for quality of life are rising as their incomes rise, but green agricultural products are also safer and healthier for consumers than traditional produce. As a result, customers are willing to spend more for them. On the other side, green agricultural products can reduce environmental pollution, conserve energy, and adhere to environmental protection policies. Because of these long-term advantages, willingness to pay a premium for the product to profit from them. Additionally, related studies (de Medeiros et al., 2016; Papista et al., 2018) demonstrate that consumers' perceptions of the value of green agricultural products influence their future purchases of green agricultural products as well as the maintenance of long-term consumer relationships (Zhuang et al., 2010; Ariffin et al., 2016). Consequently, the following study hypothesis is put out.

**H3**. Green perceived value has a significant positive effect on the willingness to pay a premium for green agricultural products.

### 2.4. Self-construal and advertising appeal have an interactional impact on the green perceived value

Self-construal, which can be further broken down into independent and interdependent self-construal, refers to the standards by which people interpret themselves, or how they perceive themselves about others when they perceive themselves (Markus and Kitayama, 1991). The self-construal theory was first applied to explain how people's values vary between cultures (Markus and Kitayama, 1991). It was then widely used to research the effectiveness of advertising and green consumer behavior. Independent self-constructed consumers, for example, are more effective when paired with individual advertising appeals, but interdependent self-constructed consumers are more effective when paired with collective advertising appeals (Chai et al., 2011). When consumers with high independent self-construals were paired with comparative advertising, advertising preferences for products with minimal cognitive engagement were more prominent (Zhang et al., 2011). Consumers with interdependent self-constructs are more inclined to put the needs of others and society above their personal needs when purchasing green products, and they are more likely to have higher purchase intentions for green agricultural products under the influence of group and societal norms (Chuang et al., 2016). Consequently, the following study hypotheses are put out.

**H4**. Advertising appeals and self-construal have an equalizing influence on the green perceived value of the environment.

**H4a**. Compared to non-green advertising appeal, there is a larger effect of matching green advertising appeal with interdependent self-construal on the green perceived value.

**H4b**. There is no appreciable difference between the effects of matching non-green advertising appeal with independent self-construal and those of green advertising appeal.

**Figure 1:**
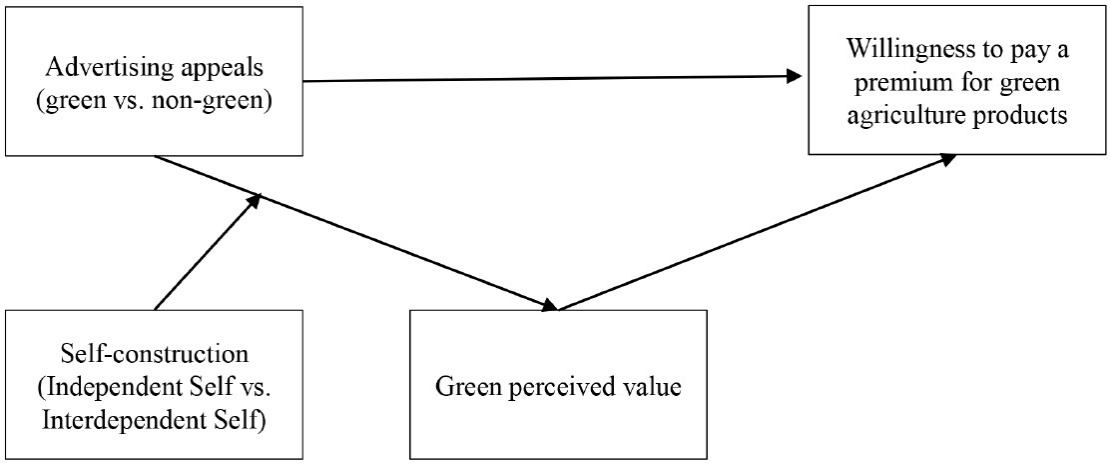
Model of the effect of advertising appeals (green vs. non-green) on willingness to pay a premium.

Combining the aforementioned analysis, the research framework is built as Figure 1.

## 3. Methodology

### 3.1. Participants and procedure

Based on the calculation method of Cohen's study and other relevant studies (Cohen, 1988; Bastian et al., 2014; Leenaars et al., 2016; Islam and Hussain, 2022), Gpower software was used to calculate the sample size of experiment 1, experiment 2, and experiment 3. The experimental effect was tested with a sample size of 159 at a significance level of 0.05 and statistical test validity of 0.80 under a medium effect size (Effect size f = 0.25), so the sample size of the subsequent experimental subjects was set to be >159.

A total of 649 data points were collected for three experiments between April and June 2022 using a questionnaire method based on the Chinese Credamo (equivalent to MTurk) questionnaire collection platform, which was well-recognized by academics (Jin et al., 2020). Participants were randomly chosen from green agricultural products consumers across all Chinese provinces. Three different sorts of screening questions were included in the questionnaire's design. The first was, “Have you ever purchased organic or green food?” The second was, “Under normal circumstances, what color is mineral water?” The third screening question, which is repeated at the beginning and end of the questionnaire, is, “Which features of the product do you think the above commercial portrays more? (again repeating the prior answer).” This was done to verify the subjects' consumption of organic produce on the one hand and to verify the accuracy of the questionnaires the subjects had to fill out on the other.

Three experiments were used in the study to experimentally analyze the hypotheses. The study used the following design to increase the veracity and usability of the experimental materials. First, for the following two reasons, all three experiments were carried out using common products that are less expensive and more familiar to consumers. On the one hand, this was done to reduce the influence of product involvement on advertising effectiveness, which is caused by the fact that the more expensive a product is, the more involved consumers are with it. On the other hand, it was done to ensure the authenticity of the experiments and the generalizability of the findings. Second, to prevent experimental bias caused by various photographs due to color and typeface, the same backdrop image was utilized for each group of studies (Wu et al., 2019). Third, the product descriptions and advertising slogans for each group of studies were taken from and modified from the offline and online advertising campaigns for the products, which were more like realistic print advertisements. Fourth, the study didn't show the product labels to customers to avoid whatever preexisting perceptions they may have had of the original produce brands (Yuan et al., 2020). Fifth, to increase the experiment's robustness and externality, different stimulus materials were employed for each of the three experiments. Sixth, the majority of the pre-experimental participants were master's and doctoral students to lessen the impact of demographic factors on the pre-experiments. Because similar group features might lessen the influence of random components (employment, wealth, education, etc.) on research findings, graduate students are frequently utilized as experimental subjects in studies relating to advertising and consumer behavior (Wang, 2014). In formal experiments, social samples are used to increase the generalizability of the research findings.

### 3.2. Measures

The questionnaire was divided into three main sections: a graphic introduction to green agricultural products; a scale for each variable; and a section with repeated screening questions and personal information about the subjects. The measurement scales for each variable were adapted from well-known research scales to accommodate the unique circumstances of the consumption of green agricultural products. Every measurement question used a Likert scale with a maximum of 7. To assure the robustness of the experimental results and multidimensional proof that the study is generalizable, different measuring scales for the same variable were used (Yuan et al., 2020).

#### 3.2.1. Willingness to pay a premium

In experiments 1 and 3, a single-item questionnaire served as the dependent variable for willingness to pay a premium (Netemeyer et al., 2004). The willingness to pay a premium question in experiment 1 was “What is the highest I would be ready to pay per 500 g for the advertised eggs, assuming the typical price of eggs in the market is 5 yuan per 500 g.” In experiment 3, the willingness to pay a premium question was “What is the highest I would be ready to pay per 500 g for the marketed apples? Assuming the typical price of apples in the market is 3 yuan per 500 g.”

A four-item questionnaire from Chaudhuri and Ligas (2009) was used to measure the dependent variable of experiment 2's willingness to pay a premium. One of the questions was, “I am willing to pay a higher price for the advertising campaign products compared to other products,” and the reliability of the willingness to pay a premium there was 0.708.

#### 3.2.2. Green perceived value

A four-item questionnaire from Yang and Zhou (2006) was used to measure the mediating variable for both experiment 2 and experiment 3. The reliability of green perceived value in experiment 2 was 0.871, and in experiment 3, the reliability of green perceived value was 0.923, and one of the questions was “I think the product of this advertising campaign will reduce environmental pollution.”

#### 3.2.3. Self-constructal

In experiment 3, the moderating variable self-construal was based on the manipulated items from Trafimow et al. (1991) and Kühnen et al. (2001). In the independent self-construal group, the guideline was “Please think carefully and write down 3 expectations you have of yourself,” while in the interdependent self-construal group, the guideline was “Please think carefully and write down 3 expectations that your family or friends have of you.”

## 4. Experimental design

Green eggs were utilized as the research subject in experiment 1 to examine the primary impact of advertising appeals (green advertising appeal vs. non-green advertising appeal) on consumers' willingness to pay a premium for environmentally friendly produce. In experiment 2, green rice was used as the research subject, and the mediating function of green perceived value was once more examined based on the main result. In experiment 3, which used green apples as its study subject, the moderating impact of self-construal was examined once more in light of the primary effect and mediating effect.

### 4.1. Experiment 1

#### 4.1.1. Pre-experiment

(1) Experimental procedure

In the simple factor design of the experiment, subjects were randomly assigned to one of two groups (advertising appeals: green vs. non-green), and images of eggs, advertising slogans, and text descriptions of the two groups of advertisements (green vs. non-green) were displayed, with consistent product images and different advertising slogans and text descriptions. This was followed by the measurement of the question items related to the manipulation of advertising appeals. In the pre-experiment, 30 volunteers were randomly divided into two experimental groups, each with 15 participants, to minimize the impact of demographic factors.

(2) Manipulation test

“Which characteristics of the product do you think the above advertisement reflects more?” was created as a validation question to check the accuracy of the manipulation. According to the results of an independent—samples *t*-test on the advertising appeals, the advertising appeal *M*_*green*_ = 1.43, SD = 1.09; *M*_*non*−*green*_ = 3.94, SD = 2.22; *p* = 0.000. The manipulation of the advertising appeals was so successful.

#### 4.1.2. Formal experiment

(1) Experimental procedure

In the simple factor design of the experiment, subjects were randomly assigned to one of two groups (advertising appeals: green vs. non-green), and images of eggs, advertising slogans, and text descriptions of the two groups of advertisements (green vs. non-green) were displayed, with consistent product images and different advertising slogans and text descriptions. It was followed by measuring the question items related to the manipulation of advertising appeals, willingness to pay a premium for green products, and demographic variables. To exclude the influence of demographic factors, 200 participants in the formal experiment were divided into two groups of 100 participants each, and after completing the questionnaire, each participant was given 1 RMB.

(2) Experimental results

Independent—samples *t*-test: willingness to pay a premium *M*_*green*_ = 8.38, SD = 3.32; *M*_*non*−*green*_ = 7.46, SD = 1.59; *p* = 0.013. Consumers' willingness to pay a premium as a result of a green advertising appeal is more significantly influenced than that of non-green advertising appeal.

Table 1 displays the findings of the descriptive statistical analysis. The sample's age distribution was 29 years on average. Females are overrepresented, most likely because they are the ones who purchase the majority of household necessities. The higher percentage of undergraduates in education, the higher education level matches with the screening question of whether they have purchased green agricultural products, probably because the education level makes this category of consumers have a higher awareness of green agricultural products. The higher percentage of income matches the likelihood that they have purchased green agricultural products.

**Table 1:** Descriptive statistical analysis of the sample in experiment 1 (*N* = 200).

**Variables**	**Definition**	**Frequency**	**Percentage (%)**
Gender	Female	131	65.50
	Male	69	34.50
Education	High school or below	10	5
	College	22	11
	Undergraduate	148	74
	Master or above	20	10
Monthly income	≤ 2,000	33	16.50
(RMB)	2,000–4,000	44	22
	4,000–6,000	41	20.50
	6,000–8,000	30	15
	≥8,000	52	26

#### 4.1.3. Discussion

The primary impact of advertising appeals (green advertising appeal vs. non-green advertising appeal) on consumers' willingness to pay a premium for green agricultural products was confirmed by experiment 1. The findings demonstrated that advertising appeals could significantly increase consumers' willingness to pay a premium for green agricultural products, with green advertising appeal eliciting a higher level of premium willingness than non-green advertising appeal. H1, H1a, and H1b were examined.

### 4.2. Experiment 2

#### 4.2.1. Pre-experiment

(1) Experimental procedure

In the simple factor design of the experiment, subjects were randomly assigned to one of two groups (advertising appeals: green vs. non-green), and images of rice, advertising slogans, and text descriptions of the two groups of advertisements (green vs. non-green) were displayed, with consistent product images and different advertising slogans and text descriptions. This was followed by the measurement of the question items related to the manipulation of advertising appeals. In the pre-experiment, 30 volunteers were randomly divided into two experimental groups, each with 15 participants, to minimize the impact of demographic factors.

(2) Manipulation test

“Which characteristics of the produce do you think the above advertisement reflects more?” was created as a validation question to check the accuracy of the manipulation. According to the results of an independent—samples *t*-test on the advertising appeals, the advertising appeal *M*_*green*_ = 2.76, SD = 1.92; *M*_*non*−*green*_ = 5.09, SD = 1.64; *p* = 0.003. The manipulation of the advertising appeals was successful.

#### 4.2.2. Formal experiment

(1) Experimental procedure

In the simple factor design of the experiment, subjects were randomly assigned to one of two groups (advertising appeals: green vs. non-green), and images of rice, advertising slogans, and text descriptions of the two groups of advertisements (green vs. non-green) were displayed, with consistent product images and different advertising slogans and text descriptions. This was followed by measurement of the question items related to the manipulation of advertising appeals, willingness to pay a premium for green agricultural products, green perceived value, and demographic variables. To exclude the influence of demographic factors, 200 participants in the formal experiment were divided into two groups of 100 participants each and were given 1 RMB after completing the questionnaire.

(2) Experimental results

Analysis of dependability shows that the questionnaire has strong reliability; the reliability of willingness to pay a premium for produce is 0.708, and the reliability of green perceived value is 0.871.

Independent—samples *t*-test: willingness to pay a premium *M*_*green*_ = 5.65, SD = 0.761; *M*_*non*−*green*_ = 5.26, SD = 0.893; *p* = 0.001. Consumers' willingness to pay a premium as a result of green advertising appeal is more significantly influenced than that of non-green advertising appeal. Green perceived value *M*_*green*_ = 6.13, SD = 0.513; *M*_*non*−*green*_=5.39, SD = 1.10; *p* = 0.000. Consumers' influence on the green perceived value as a result of green advertising appeal is more significantly influenced than that of non-green advertising appeal.

**Figure 2:**
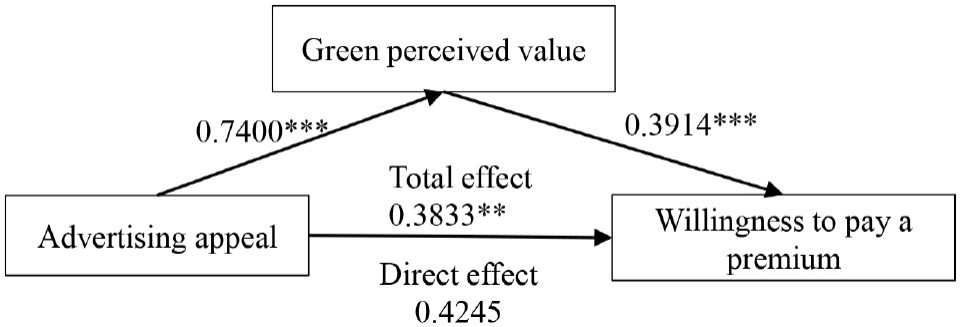
Analysis of mediating effect.

Figure 2 displays the findings of the mediating effect analysis. To examine the relationship between advertising appeals and consumers' willingness to pay a premium for green agricultural products, PROCESS model 4 was utilized. Results with a sample size of 5,000 and 95% confidence interval revealed that the indirect effect was significant (β = 0.2896, 95% CI = [0.160,0.419]) and the direct effect was not significant(β = 0.2896, 95% CI = [0.160,0.419]). The total effect of advertising appeals on willingness to pay a premium for green agricultural products was significant (β = 0.3833, *p* = 0.0013, 95% CI = [0.1520,0.6147]). As a result, advertising appeals and willingness to pay a premium for organic produce are full mediated by green perceived value.

Table 2 displays the findings of the descriptive statistical analysis. The sample's age distribution was 30.5 years on average. Other sample characteristics matched those discovered for the samples from experiment 1.

**Table 2:** Descriptive statistical analysis of the sample in experiment 2 (*N* = 200).

**Variables**	**Definition**	**Frequency**	**Percentage (%)**
Gender	Female	111	55.50
	Male	89	44.50
Education	High school or below	8	4
	College	16	8
	Undergraduate	153	76.50
	Master or above	23	11.50
Monthly income	≤ 2000	13	6.50
(RMB)	2,000–4,000	35	17.50
	4,000–6,000	30	15
	6,000–8,000	37	18.50
	≥8,000	85	42.5

#### 4.2.3. Discussion

Experiment 2 confirmed the mediating effect of green perceived value. as well as the main influence of advertising appeals (green advertising appeal vs. non-green advertising appeal) on the willingness to pay a premium for green agricultural products. The findings demonstrate that advertising appeal can significantly increase consumers' willingness to pay more for green agricultural products than non-green advertising appeal, can significantly increase consumers' green perceived value relative to non-green advertising appeal, and can significantly increase consumers' willingness to pay a premium for green agricultural products. H2 and H3 were confirmed, while H1, H1a, and H1b were confirmed once more.

### 4.3. Experiment 3

#### 4.3.1. Pre-experiment

(1) Experimental procedure

To once more confirm the main and mediating effects as well as the moderating influence of self-construal, materials were used to stimulate subjects' contextual self-construal at the start of the experiment. The study applied a 2 (green advertising appeal vs. non-green advertising appeal) ×2 (self-construal: interdependent self vs. independent self) two-factor intergroup randomized experiment, in which participants were randomly assigned to four experimental groups. Self-construal and advertising appeals manipulation question items were measured *post hoc*, and the product visuals were the same for all four groups but with various advertising slogans and textual descriptions. The pre-60 experiment's participants were divided into four experimental groups, with 15 participants in each, to eliminate the influence of demographic factors.

(2) Manipulation test

The validity of the self-construal manipulation was tested by asking, “Did the thinking just now make me think of myself or my family and friends?” The independent sample t-test on the self-construal yielded the following results: *M*_*independent*_ = 2.83, SD = 2.12; *M*_*interdependent*_ = 4.67, SD = 2.19; *p* = 0.002. Consequently, the manipulation of the advertising appeal proved successful.

The validity of the advertising appeals manipulation was tested by asking, “Which attribute of the product do you think the above advertisement reflects more?” The findings of an independent sample *t*-test on the advertising appeals revealed that the advertising appeal *M*_*green*_ = 2.57, SD = 1.70; *M*_*non*−*green*_ = 5.03, SD = 1.97; *p* = 0.000. Consequently, the manipulation of advertising appeals proved successful.

#### 4.3.2. Formal experiment

(1) Experimental process

The formal experiment will have 249 participants, with ~62 in each group. After completing the questionnaire, each participant will receive a reward of 1RMB. The experimental procedure is the same as the pre-experiment.

(2) Experimental results Analysis of dependability:

The questionnaire had a strong level of reliability, as evidenced by the reliability of the green perceived value, which was 0.923. Independent samples *t*-test: Willingness to pay a premium *M*_*green*_ = 5.35, SD = 1.234; *M*_*non*−*green*_=4.90, SD = 0.968; *p* = 0.001. Consumers' willingness to pay a premium as a result of green advertising appeal is more significantly influenced than that of non-green advertising appeal. Green perceived value *M*_*green*_ = 6, 02, SD = 0, 734; *M*_*non*−*green*_ = 4.65, SD = 1.346; *p* = 0.000. Consumers' influence on the green perceived value as a result of green advertising appeal is more significantly influenced than that of non-green advertising appeal.

Mediating effect analysis: using the Jamovi program, an examination of the relationship between advertising appeals and willingness to pay a premium for green agricultural products was undertaken. The findings indicated that the indirect effect was significant (β = 0.277, *p* = 0.038, 95% CI = [0.0098,0.360]), the direct effect was not significant (β = 0.1196, *p* = 0.100, 95% CI = [−0.0515, 0.590]), and the overall effect of advertising appeals on willingness to pay a premium for green agricultural products was significant (β = 0.2018, *p* = 0.001, 95% CI = [0.1797, 0.728]). Then, advertising appeals and willingness to pay a premium for green agricultural products were full mediated by green perceived value. The same outcomes as in experiment 2.

**Figure 3:**
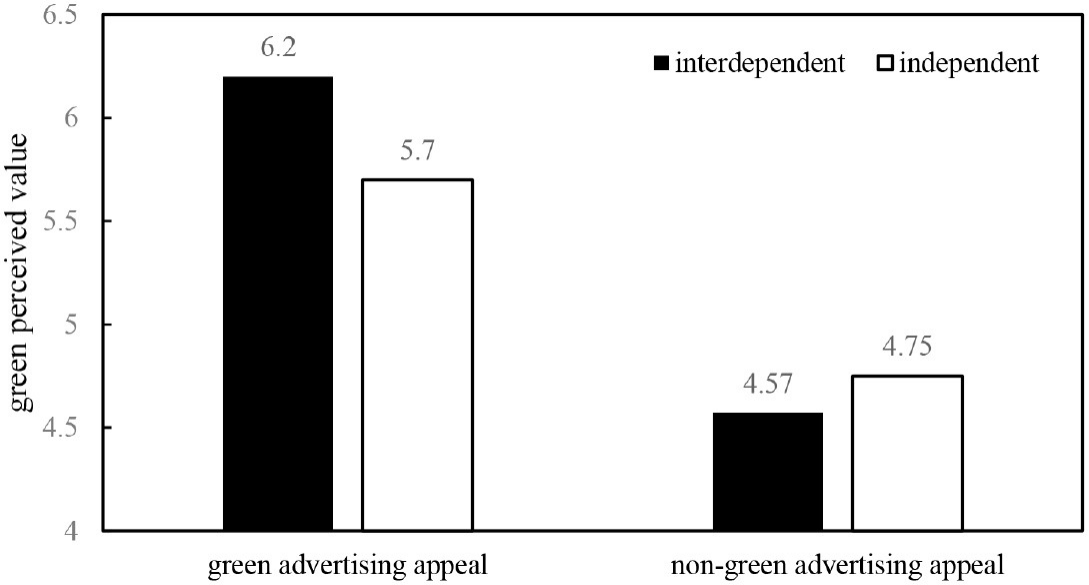
The interaction effect of self-construal and advertising appeals on the green perceived value.

Interactive effect analysis: To analyze the interactive effect of self-construal on the relationship between advertising appeals and green perceived value, Jamovi was utilized. Table 3 displays the ANOVA results and demonstrates how advertising appeals have a positive impact on green perceived value. The Interactive effect between advertising appeals and self-construal is also found to be significant, supporting H4 as well. Figure 3's simple effects analysis findings revealed that consumers in the green advertising appeal group had higher levels of green perceived value with interdependent self matching (*M*_*interdependent*_ = 6.2, *M*_*independent*_ = 5.7, *p* = 0.000, supporting H4a). The effect of matching non-green advertising appeal to self-construal on green perceived value did not differ significantly from that of non-green advertising appeal (*M*_*interdependent*_ = 4.57,*M*_*independent*_ = 4.75, *p* = 0.464, supporting H4b).

**Table 3:** ANOVA results.

	**SS**	**F**	***p***
Model	124.55	35.65	<0.001
Advertising appeals	99.89	85.77	<0.001
Self-construal	1.55	1.33	0.25
Advertising appeals * Self-construal	6.73	5.78	0.017

Table 4 displays the findings of the descriptive statistical analysis. The sample's age distribution was 29.9 years on average. Other sample characteristics matched those discovered for the samples from experiment 1.

**Table 4:** Descriptive statistical analysis of the sample in experiment 3 (*N* = 249).

**Variables**	**Definition**	**Frequency**	**Percentage (%)**
Gender	Female	160	64.30
	Male	89	35.70
Education	High school or below	11	4.40
	College	25	10
	Undergraduate	170	68.30
	Master or above	43	17.30
Monthly income	≤ 2000	36	14.50
(RMB)	2,000–4,000	43	17.30
	4,000–6,000	44	17.70
	6,000–8,000	50	20.10
	≥8,000	76	30.50

#### 4.3.3. Discussion

The three effects of self-construal were once more confirmed in experiment 3, along with their mediating and moderating effects. The study's findings indicate that advertisements can significantly increase consumers' willingness to pay a premium for green agricultural products, with green advertising appeal having a higher likelihood of doing so than non-green advertising appeal. The connection between advertising appeals and willingness to pay a premium is moderated by green perceived value. Advertising appeals and green perceived value interacted with each other through self-construal, and consumers who were exposed to green advertising appeal had higher levels of green perceived value when their interdependent selves were matched. Comparing the impact of green advertising appeal to non-green advertising appeal on the green perceived value, there was no discernible difference. H4, H4a, and H4b were confirmed, and H1, H1a, H1b, H2, and H3 underwent new testing.

## 5. Conclusions and implications

### 5.1. Research findings and discussion

First, the willingness to pay a premium for green agricultural products was significantly influenced by advertising appeals. A substantial willingness to pay a premium was more likely to result in green advertising appeal than non-green advertising appeal. This finding differs from previous research on products with low environmental impact (cereals) in two ways (Kong and Zhang, 2014). One is that a previous study concluded that there was no difference in the impact of green advertising appeal and non-advertising green appeal on willingness to pay a premium, while this study concluded that green advertising appeal is more likely to make consumers more willing to pay a premium than non-green advertising appeal, which may have two reasons, on the one hand, because the subjects of this study are eggs, rice, and apples, respectively, which are more concrete products than simple cereals, and previous studies showed that concrete information was more likely to make consumers more willing to buy than abstract information (Xue et al., 2019). On the other hand, it may be because cereals simply indicate their slogans (Jiang et al., 2021), while the products in this study, in addition to their slogans, have a green food mark that is recognized and licensed by specialized agencies, which is more recognized and trusted by consumers than other products, and consumers are willing to pay extra for this certification. The second is that most prior research has been done from the perspective of other types of advertising appeals on willingness to pay (Wang, 2017; Wang et al., 2017; Li and Sun, 2022). However, this study's willingness to pay a premium refers to consumers' willingness to pay for the same type of product when they are aware that A's price is higher than B's, but consumers are still willing to pay the extra price (Netemeyer et al., 2004). When compared to the willingness to pay, willingness to pay a premium is one of the best predictors of consumer loyalty (Aaker, 1996) and can more accurately depict consumers' actual behavior (Netemeyer et al., 2004). In comparison to earlier research, this one shows a stronger explanatory power in terms of variable selection.

Second, consumers' green perceived value is more strongly influenced by green advertising than by non-green advertising, with green advertising acting as a full mediator between advertising appeals and the willingness to pay a premium for green agricultural products. The following is where the results of this study diverge from those of earlier ones. The first distinction is that while fewer researchers have examined the full mediating role played by green perceived value, existing studies are more likely to analyze the partial mediating role played by the higher-order variable of perceived value (Wang, 2017; Wang et al., 2017; Li and Sun, 2022). This study aims to advance the research on perceived value and offer suggestions for future research on green perceived value. The second distinction is that most existing studies analyze purchase intention from the perspective of emotional appeal and altruistic advertising appeal (Jaeger and Weber, 2020), but less from the perspective of green and non-green advertising appeals. The findings suggest that the green perceived value of consumers' produce directly affects consumers' willingness to pay a premium and that the higher the green value of produce perceived by consumers from advertising appeals, the higher the consumer loyalty (Chen, 2013), which in turn encourages consumers to pay higher prices for both green and non-green advertising appeals.

Third, self-construal played an interactive role between advertising appeals and green perceived value. This finding differs from the existing studies in two ways. The first point is that existing studies more often analyze self-construal as a moderating role between advertising appeals and purchase intention (Kareklas et al., 2014; Yang et al., 2015; Hornik et al., 2017), while this study analyzed the effect of the interaction between self-construal and advertising appeals on green perceived value, with different dependent variables, which helped broaden self-construal related research. The second point is that most of the existing studies on self-construal and advertising appeals classified advertising appeals as emotional appeal/rational appeal, personal nostalgia appeal/historical nostalgia appeal, and charity advertising (Hong and Chang, 2015; Chang and Feng, 2016; Xu, 2019; Lee et al., 2020), and this study analyzed from the green/non-green perspective, which helped to improve the theoretical model related to advertising appeals. The findings showed that consumers' value differences and self-construal affected the effectiveness of advertising appeals, and when consumers' interdependent self-construal was dominant, consumers paid more attention to the social and overall interests reflected in advertising appeals, and green advertising appeal was more consistent with interdependent self-construal, and advertising messages were more easily accepted by consumers (Mao et al., 2017). Green advertising appeal was more persuasive and had higher green perceived value.

### 5.2. Theoretical contributions

First, it made clear how advertising appeals affected consumers' willingness to pay a premium for green agricultural products. Although studies had shown that advertising appeals had a significant positive effect on the academic's less well-understood willingness to pay a premium for green agricultural products, the mechanism of this study. To start, advertising appeals were typically divided into emotional/rational appeals, egoistic/altruistic appeals, and concrete/abstract appeals (Kareklas et al., 2014; Yang et al., 2015; Hornik et al., 2017), and were less frequently analyzed from a green/non-green. Next, analyses focused more on click-through rate, word-of-mouth, customer response, and readiness to buy than on willingness to pay a premium (Green and Peloza, 2014; Kim et al., 2020; Lu et al., 2021). There were fewer assessments from the standpoint of green agricultural products, and the majority of earlier studies focused on batteries, paper towels, beverages, laundry detergents, and autos (Schuhwerk and Lefkoff-Hagius, 1995; Kong and Zhang, 2014; Yang et al., 2015; Lu et al., 2021). Since they had no purpose other than to indirectly harm customers' health, research on green agricultural products was required (Ottman, 2010).

Second, expand the study of green perceived value as a mediating factor. To begin with, fewer studies have been done from the standpoint of green perceived value, and more studies have focused on perceived value as a higher-order variable (Xue et al., 2019; Cao et al., 2021). Next, the existing articles that examine the impact of advertising appeals on perceived green value typically draw from appeals to self-interest, altruism, and advertising intrinsic goals vs. extrinsic goals (Li and Cui, 2021; Li and Sun, 2022). The previous research has more frequently examined the impact of perceived value on the willingness to pay for green products and less frequently examined the impact of perceived value on the willingness to pay a premium for produce. Furthermore, less research has been done from the perspective of produce, with the influence of perceived value on green product purchase intention mostly examined for consumers of clothes, housing, tourism, and all e-commerce (Wang et al., 2017; Li and Sun, 2022). The study contributes to the body of knowledge about green perceived value as a mediating factor in the relationship between advertisement attractiveness and willingness to pay a premium.

### 5.3. Managerial implications

First, when engaging in green marketing, businesses can emphasize the distinctive qualities of green agricultural products, such as their safety and health, environmental friendliness, etc., to further differentiate them from conventional produce. They can also highlight the differentiated benefits of green agricultural products, encourage consumers to recognize the green attributes, cater to their needs, and ultimately persuade customers to purchase green agricultural products.

Second, to increase the effectiveness of advertising appeals, enterprizes should raise consumers' perceptions of the value of green products by providing them with advertising information while shopping. The green perceived value acts as a mediator between advertising appeals and willingness to pay a premium for green agricultural products. To increase customers' green perceived value and ultimately increase their willingness to pay premium, images, music, and films about the green value of green agricultural products can be played during the advertising and marketing process.

Third, the effectiveness of corporate advertising appeals might be impacted by the self-constructed attributes of consumers. Businesses can utilize digital tools to gather and evaluate consumer personality attributes before making targeted appeals to various consumer traits. Businesses can increase the potency of their advertising appeals by using the personal pronoun technique, task activation method, and story activation method in their campaigns to temporarily activate consumers' interdependent self-construal.

### 5.4. Inadequate investigation

First, the experimental materials consist of pictures, advertising slogans, and product introductions with no video display, which will somewhat affect the communication effect of advertising and is more appropriate for the promotion of green agricultural products that are sold offline, though some produce are sold online with the use of video communication tools.

Second, the willingness to pay a premium is primarily assessed using a variety of scales. Consumer self-assessment may be biased, therefore the follow-up study will focus on actual observational data points such as click-through rate and purchase frequency.

## Data availability statement

The raw data supporting the conclusions of this article will be made available by the authors, without undue reservation.

## Author contributions

Conceptualization, validation, writing—original draft preparation, and visualization: MZ. Methodology, software, formal analysis, and writing—review and editing: DT. Investigation and data curation: JC. Supervision: AX and QZ. Funding acquisition: QZ. All authors have read and agreed to the published version of the manuscript.

## Funding

This research was funded by Introduction of Talents of Minjiang University Science and Technology Pre-research Project Research on The Impact of Green Advertising Appeals on Consumers' Willingness to Pay Premium for Green Agricultural Products and Major Project of Social Science Fund of Fujian Province in 2022 Research on the Strategy of Fujian Province Adhering to Expanding Domestic Demand.

## Conflict of interest

The authors declare that the research was conducted in the absence of any commercial or financial relationships that could be construed as a potential conflict of interest.

## Publisher's note

All claims expressed in this article are solely those of the authors and do not necessarily represent those of their affiliated organizations, or those of the publisher, the editors and the reviewers. Any product that may be evaluated in this article, or claim that may be made by its manufacturer, is not guaranteed or endorsed by the publisher.
